# ^13^C-detected NMR experiments for automatic resonance assignment of IDPs and multiple-fixing SMFT processing

**DOI:** 10.1007/s10858-015-9932-9

**Published:** 2015-04-23

**Authors:** Paweł Dziekański, Katarzyna Grudziąż, Patrik Jarvoll, Wiktor Koźmiński, Anna Zawadzka-Kazimierczuk

**Affiliations:** Faculty of Chemistry, Biological and Chemical Research Centre, University of Warsaw, Żwirki i Wigury 101, 02-089 Warsaw, Poland; Faculty of Physics, University of Warsaw, Pasteura 5, 02-093 Warsaw, Poland; Agilent Technologies, 10 Mead Road, Yarnton, OX5 1QU UK

**Keywords:** Intrinsically disordered proteins, ^13^C direct-detection NMR, High-dimensional NMR experiment, Non-uniform sampling, Automatic assignment, Sparse multidimensional Fourier transform

## Abstract

Intrinsically disordered proteins (IDPs) have recently attracted much interest, due to their role in many biological processes, including signaling and regulation mechanisms. High-dimensional ^13^C direct-detected NMR experiments have proven exceptionally useful in case of IDPs, providing spectra with superior peak dispersion. Here, two such novel experiments recorded with non-uniform sampling are introduced, these are 5D HabCabCO(CA)NCO and 5D HNCO(CA)NCO. Together with the 4D (HACA)CON(CA)NCO, an extension of the previously published 3D experiments (Pantoja-Uceda and Santoro in J Biomol NMR 59:43–50, 2014. doi:10.1007/s10858-014-9827-1), they form a set allowing for complete and reliable resonance assignment of difficult IDPs. The processing is performed with sparse multidimensional Fourier transform based on the concept of restricting (fixing) some of spectral dimensions to a priori known resonance frequencies. In our study, a multiple-fixing method was developed, that allows easy access to spectral data. The experiments were tested on a resolution-demanding alpha-synuclein sample. Due to superior peak dispersion in high-dimensional spectrum and availability of the sequential connectivities between four consecutive residues, the overwhelming majority of resonances could be assigned automatically using the TSAR program.

**Electronic supplementary material:**

The online version of this article (doi:10.1007/s10858-015-9932-9) contains supplementary material, which is available to authorized users.

## Introduction

Intrinsically disordered proteins (IDPs) have recently attracted much interest of researchers studying biological mechanisms (Oldfield and Dunker 2014). Notably, ca. 25–30 % of proteins found in Eukaryota are intrinsically disordered (Oldfield et al. 2005). IDPs are extremely flexible and reveal only transient secondary structure, which allows them to play many important roles in living organisms (Wright and Dyson 1999), often connected with signaling and regulation processes. They are also associated with many human diseases, like cancer (Iakoucheva et al. 2002) or neurodegenerative diseases (Uversky and Fink 2004). Much effort has been recently put into improving experimental techniques of studying IDPs. Importantly, X-ray crystallography that is often considered to be the leader in structural proteomics cannot be applied. High mobility of the polypeptide chain usually prevents crystallization, which is essential for X-ray crystallography. On the contrary, NMR provides both structural and dynamic information with atomic resolution. It allows determining the conformational propensities or finding the regions involved in ligand binding. This makes NMR the main tool for studying IDPs.

The first step of every NMR-based protein research is a sequence-specific assignment of resonances. A large variety of experiments appropriate for assignment of IDPs is available (Kazimierczuk et al. 2010; Mäntylahti et al. 2011; Zawadzka-Kazimierczuk et al. 2012b; Solyom et al. 2013; Pantoja-Uceda and Santoro 2013; Liu and Yang 2013; Hellman et al. 2014; Pantoja-Uceda and Santoro 2014; Reddy and Hosur 2014; Piai et al. 2014; Yao et al. 2014; Yoshimura et al. 2015). Among them ^13^C direct-detected high-dimensional (≥4D) techniques (Nováček et al. 2011, 2012, 2013; Bermel et al. 2012, 2013a) are especially useful, due to their specific features described below.

The high dimensionality of the experiments gives the possibility to circumvent the problem of poor dispersion of chemical shifts, caused by high flexibility of IDP backbone. However, the high flexibility of IDPs has also a positive aspect—it results in reduced transverse relaxation rates when compared to structured proteins of similar size. Thus, even long pulse sequences (employed in high-dimensional experiments) are still practical.

^13^C detection is helpful if amide protons (typically used for NMR signal detection) undergo fast chemical exchange. It also allows efficient assignment of proline-rich proteins. Proline residue does not give a signal in HN-detected spectra, creating a break in chains of sequentially-linked residues. Notably, prolines, as disorder-promoting residues, are more abundant in IDPs than in folded proteins (Dunker et al. 2008). Another benefit of carbon detection is that it takes an advantage of peak dispersion in CO dimension that is superior to HN, usually used for NMR signal detection.

High-dimensional and ^13^C-detected experiments require special approach to data acquisition and processing. As the linewidths are inversely proportional to maximum evolution times (in each spectral dimension), conventional sampling of the evolution time space generally does not provide the sufficiently narrow peaks for ≥4D experiments within practically achievable experiment duration. This is caused by the fact that the distance between the equally-distanced sampling points is defined by the Nyquist theorem (Nyquist 2002). Therefore, to acquire high-dimensional data, non-uniform sampling (NUS) of the evolution-time space should be employed. For that, one has to choose a method of processing of such data: projection reconstruction (Freeman and Kupče 2012), automated projection spectroscopy, APSY (Hiller and Wider 2012), maximum entropy (Mobli and Hoch 2008), multi-way decomposition (Malmodin and Billeter 2005), multi-dimensional decomposition (Orekhov and Jaravine 2011), multidimensional Fourier transform (Kazimierczuk et al. 2012), compressed sensing (Holland and Gladden 2014). Finally, there remains an important problem of handling the high-dimensional spectrum. No software for displaying 5D and higher-dimensional spectra is so far available. Moreover, the full-dimensional spectrum would be a very large file (tens GB and more). Thus, usually the full-dimensional spectrum is not calculated. Some methods come with a software for automatic analysis of high-dimensional data [APSY–GAPRO (Hiller et al. 2005), multiway decomposition—PRODECOMP (Malmodin and Billeter 2006)], without giving access to a full spectrum. Another approach is calculating just the regions of the spectra that contain peaks (without losing any information), as in sparse multidimensional Fourier transform, SMFT (Kazimierczuk et al. 2009).

Despite all the aforementioned advantages, ^13^C-detection is also associated with some experimental limitations. Firstly, ^13^C detection features lower sensitivity than ^1^H detection. This is primarily caused by the difference in gyromagnetic ratios of ^1^H and ^13^C nuclei. The problem has been alleviated by constant improvement in NMR instrumentation, including ^13^C-detection optimized cryogenically cooled probes. Another problem of carbon-detected experiments is peak splitting caused by the large value of one-bond homonuclear C–C scalar coupling. This effect can be avoided by applying band-selective carbon homodecoupling during acquisition or so called virtual decoupling (Bermel et al. 2006a).

Taking advantage of the methods presented above (NUS, SMFT, virtual decoupling), several high-dimensional ^13^C-detected experiments were recently elaborated. To establish the sequential connectivities between the residues, some of the experiments use aliphatic chemical shifts (HA_i_–CA_i_ and/or HB_i_–CB_i_ pairs), others—better resolved and thus more efficient CO_i−1_–N_i_ pairs. Some of the techniques employ amide proton excitation, other excite alpha protons preserving proline signals. All the previously proposed techniques establish the sequential connectivities between two adjacent residues.

In the current study we present two 5D ^13^C-detected experiments. They are combined with the 4D (HACA)CON(CA)NCO experiment, an extension of the previously published 3D hacaCOncaNCO and 3D hacacoNcaNCO experiments (Pantoja-Uceda and Santoro 2014). The 4D (HACA)CON(CA)NCO experiment serves as a basis spectrum for SMFT processing. This allows us to calculate spectral cross-sections in several ways, which we refer to as multiple fixing. As a result, 4D (HACA)CON(CA)NCO spectrum provides the sequential connectivities (via CO_i−1_–N_i_ pairs) between four consecutive residues, allowing to resolve even difficult cases with high degree of chemical shift degeneracy. 5D spectra provide additionally amide proton (HNCO(CA)NCO spectrum) and aliphatic HA, CA, HB, CB (HabCabCO(CA)NCO spectrum) chemical shifts. Moreover both 5D techniques provide sequential connectivities between two adjacent residues. The set of experiments allows for efficient automatic assignment of IDPs’ resonances.

## Materials and methods

A sample of 1.2 mM ^13^C,^15^N-uniformly labeled alpha-synuclein protein (purchased from Giotto Biotech) in 20 mM sodium phosphate buffer, at pH 6.5 was used for all experiments. The experiments were performed at the temperature of 298 K, on Agilent NMR system spectrometer operating at 599.6 MHz ^1^H, 150.8 MHz ^13^C and 60.8 MHz ^15^N resonance frequencies. The spectrometer was equipped with a HCN ^13^C-enhanced cryogenic probe. High-power ^1^H, ^13^C and ^15^N π/2 pulses of 11.35, 13.2 and 29.5 µs, respectively, were used. Selective CA and CO pulses were realized as phase-modulated (for off-resonance excitation or inversion) *sinc* shapes, with B1 field strength adjusted to have a minimal effect on CO and CA, respectively. Simultaneous inversion of CA and CO spins was achieved using six-element composite pulse (Shaka 1985). Decoupling of ^1^H was achieved with waltz16 sequence (Shaka et al. 1983). All gradients employed had 500 µs of duration and were applied along the z axis. Each experiment was acquired in a pseudo-2D mode, with the States-TPPI method applied in all indirect dimensions to achieve quadrature detection. All experiments employed the IPAP approach (Bermel et al. 2006b, 2008) to avoid peak splitting in the direct dimension caused by the CA–CO scalar couplings. The in-phase (IP) and anti-phase (AP) components were acquired and stored in an interleaved manner, doubling the number of FIDs recorded (Bermel et al. 2006a). In all experiments four scans per increment were acquired. The acquisition time was set to 106 ms and relaxation delay to 1 s. The experiments were performed using random on-grid Poisson disk sampling with sampling density set according to a Gaussian distribution exp(−(t/0.5)^2^) with regard to maximum evolution time (Kazimierczuk et al. 2008). The time schedules were generated using the *RSPack* program available from http://nmr.cent3.uw.edu.pl/software. The numbers of indirectly-detected time points, maximum evolution times and spectral widths in indirect dimensions are shown in Table 1. The pulse sequences were written for Agilent spectrometers using own-developed programming library and are available from the authors upon request.

**Table 1:** Experimental parameters for indirect dimensions of all experiments

Experiment	ni^a^	Measurement time	Dimension 1			Dimension 2			Dimension 3			Dimension 4		
			nucl	t_max_^b^	sw^c^	nucl	t_max_^b^	sw^c^	nucl	t_max_^b^	sw^c^	nucl	t_max_^b^	sw^c^
4D (HACA)CON(CA)NCO	2400	53 h 15 min	CO	28	2.2	N	28	2.8	N	28	2.8		n.a.	
5D HabCabCO(CA)NCO	1800^d^	78 h 40 min^d^	Hab	10	6	Cab	7.1	15	CO	28	2.2	N	28	2.8
5D HNCO(CA)NCO	1800^e^	81 h 10 min^e^	HN	10	5.2	N	28	2.8	CO	28	2.2	N	28	2.8

^a^Number of increments in all indirectly-detected dimensions together

^b^Maximum evolution time in indirectly-detected dimension, ms

^c^Spectral width in indirectly-detected dimension, kHz. The spectral widths were set in a safe manner. Unlike in conventional experiments, in NUS experiments the measurement time is very weakly dependent on the spectral widths and maximum evolution time achieved, so too wide ranges do not significantly influence quality of the spectra

^d^Identical result could be obtained using 1200 increments (the measurement time would be then 52.5 h)

^e^Identical result could be obtained using 1200 increments (the measurement time would be then c.a. 54 h)

The multidimensional Fourier transform (MFT) (Kazimierczuk et al. 2006), implemented in the *ToASTD* program, was used for processing of the basis 4D (HACA)CON(CA)NCO experiment. The sparse multidimensional Fourier transform (SMFT) (Kazimierczuk et al. 2009), implemented in the *reduced* program, with ‘fixed’ frequencies derived from the basis spectrum peak list, was used to obtain 2D cross-sections of all experiments. Both *ToASTD* and *reduced* programs are available from http://nmr.cent3.uw.edu.pl/software. Prior to direct-dimension processing, both programs performed the appropriate handling of in-phase and anti-phase components so that no doublets appeared in the spectrum. For processing of directly detected dimension, cosine square weighting function was used prior to Fourier transform with zero-filling to 2048 complex points. No apodization was applied in indirect dimensions.

The resulting spectra were displayed using the SPARKY software (Goddard and Kneller 2002). The assignment of protein resonances was done automatically using the TSAR program (Zawadzka-Kazimierczuk et al. 2012a), available from http://nmr.cent3.uw.edu.pl/software. The TSAR input files in which the new experiment types are defined are shown in Supplementary Materials.

## Results and discussion

### SMFT processing

As stated above, handling of high-dimensional spectra is problematic due to large file size and lack of appropriate software for displaying the high-dimensional spectra. Furthermore, such spectra are mostly empty: in multidimensional space (of several hundred frequency points in each dimension) typically just several hundred peaks are present (few peaks per residue). The sparse multidimensional Fourier transform, SMFT (Kazimierczuk et al. 2009), allows to limit the processing to the regions that contain peaks. The information on the location of the regions is gathered by recording certain basis spectrum that shares some dimensions with the high-dimensional spectrum. Thus, for each peak of the basis spectrum, one (or more) cross-section(s) of the high-dimensional spectrum can be calculated, by fixing the frequencies obtained from the basis peak list. Up to now, the basis spectrum was chosen in a way that allowed to calculate just a single cross-section for each peak (Kazimierczuk et al. 2009, 2010; Nováček et al. 2011; Zawadzka-Kazimierczuk et al. 2012b; Bermel et al. 2013a). In the present study, we chose the basis spectrum in a way that allows us to calculate a few different cross-sections for each basis peak. Such multiple fixing, will make it possible to do an easy expansion of the spin systems and thus facilitates the establishment of sequential connectivities.

### Experimental techniques and data processing

We developed a set of ^13^C-detected high-dimensional techniques, consisting of 4D (HACA)CON(CA)NCO, 5D HabCabCO(CA)NCO and 5D HNCO(CA)NCO. It allows the assignment of the following resonances: HN, N, CO, CA, CB, HA and HB. However, not always all of these experiments are needed to obtain the resonance assignment. As shown below, the 5D HNCO(CA)NCO experiment can be excluded and still the assignment can be obtained. All of these experiments are recorded using NUS, which allows to achieve extraordinary resolution in a reasonable experimental time. The first two of the above experiments exploit excitation of aliphatic protons, allowing preservation of proline signals. The last one, 5D HNCO(CA)NCO, excites amide protons and is the only one which yields amide proton chemical shifts. In all three techniques the signal is acquired on carbon channel, and the detected nuclei are carbonyl carbons.

4D (HACA)CON(CA)NCO is processed with multidimensional Fourier transform, MFT (Kazimierczuk et al. 2006). The obtained 4D spectrum serves as the basis spectrum during SMFT processing. Two other experiments are processed with sparse multidimensional Fourier transform, SMFT (Kazimierczuk et al. 2009). For such spectra the multiple fixing method can be applied. Interestingly, also the basis spectrum can be SMFT-processed (also with multiple fixing), basing on itself (see below).

**Fig. 1:**
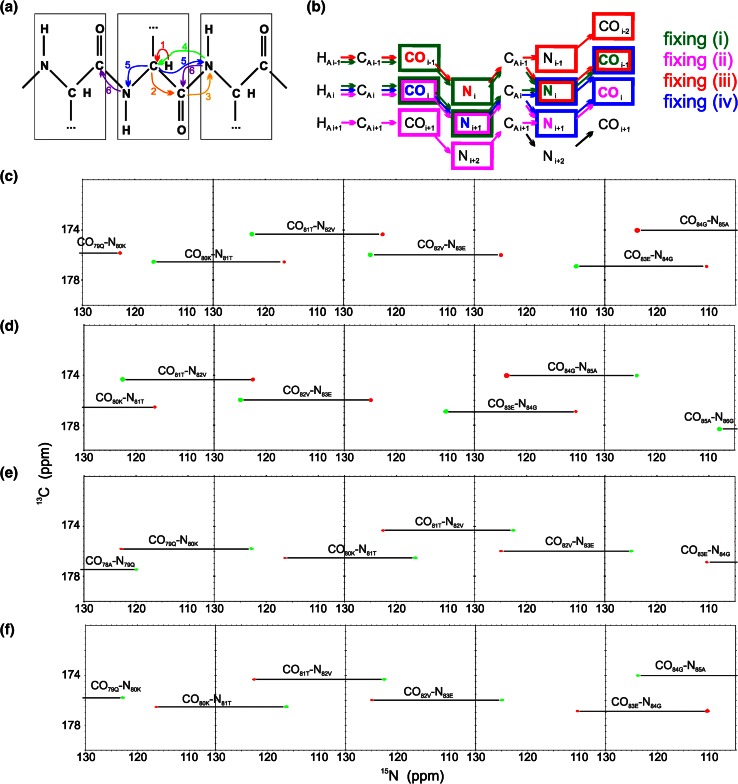
4D (HACA)CON(CA)NCO technique. **a** Coherence transfer in a polypeptide chain. **b** Four ways of fixing the frequencies for SMFT processing, shown using *different colors* on a coherence transfer scheme: (*1i*)—*green*, (*1ii*)—*magenta*, (*1iii*)—*red*, (*1iv*)—*blue* (*different fixing symbols* are explained in the text). Highlighting the text with an appropriate *color* shows the fixed frequencies used during SMFT, the frequencies appearing on a cross-section are marked using frames of the *same color*. **c**–**f** 2D cross-sections of the 4D spectrum of alpha-synuclein protein, obtained by SMFT procedure, using (*1i*) fixing—panel (**c**), (*1ii*) fixing (**d**), (*1iii*) fixing (**e**) and (*1iv*) fixing (**f**). The cross-sections correspond to the 80K–84G protein fragment. Cross-sections corresponding to the same basis peak are placed one over another

4D (HACA)CON(CA)NCO is an extension of the previously published 3D versions of this experiment: hacaCOncaNCO and hacacoNcaNCO (Pantoja-Uceda and Santoro 2014). The pulse sequence is shown in Fig. S1 and magnetization transfer pathway is shown in Fig. 1a. The numbering of experimental dimensions (used below) is the following: (HACA)CO^1^N^2^(CA)N^3^CO^4^ (dimension 4 is directly detected). The 4D spectrum contains peaks of two types: CO_i_–N_i+1_–N_i_–CO_i−1_ (negative intensity unless residue *i* is glycine) and CO_i_–N_i+1_–N_i+1_–CO_i_ (positive intensity unless residue *i* is glycine). The latter peak type is diagonal and it contains no new information with respect to the former peak type. Thus, just the peaks of the former type are incorporated into the basis peak list. Once the basis peak list is ready, the same data can be processed with SMFT. As the experiment’s dimensionality is four, two frequencies have to be fixed to calculate a 2D cross-section. These two frequencies can be chosen (from the basis peak list containing CO_i_–N_i+1_–N_i_–CO_i−1_ peaks) in four different ways (Fig. 1b): (1i) dimension 3 fixed based on N_i_ frequencies and dimension 4 fixed on CO_i−1_ frequencies: on such a cross-section peaks of two types appear: CO_i−1_–N_i_ and CO_i_–N_i+1_, (1ii) dimension 3 fixed based on N_i+1_ frequencies and dimension 4 fixed on CO_i_ frequencies: on such a cross-section peaks of two types appear: CO_i_–N_i+1_ and CO_i+1_–N_i+2_, (1iii) dimension 2 fixed based on N_i_ frequencies and dimension 1 fixed on CO_i−1_ frequencies: on such a cross-section peaks of two types appear: CO_i−2_–N_i−1_ and CO_i−1_–N_i_, (1iv) dimension 2 fixed based on N_i+1_ frequencies and dimension 1 fixed on CO_i_ frequencies: on this cross-section peaks of two types appear: CO_i−1_–N_i_ and CO_i_–N_i+1_. Versions (1i) and (1ii) include direct dimension fixing, while (1iii) and (1iv) do not. To calculate versions (1i) and (1iv), different basis frequencies are used, nonetheless both versions yield identical peak types. Therefore they can be used to resolve overlapping cross-sections (see “The strategy of resonance assignment and multiple fixing method” section). Altogether, in all cross-sections corresponding to a single basis peak, four consecutive CO–N pairs are present. An example of 2D cross-sections obtained from 4D (HACA)CON(CA)NCO experiment is shown in Fig. 1c–f.

**Fig. 2:**
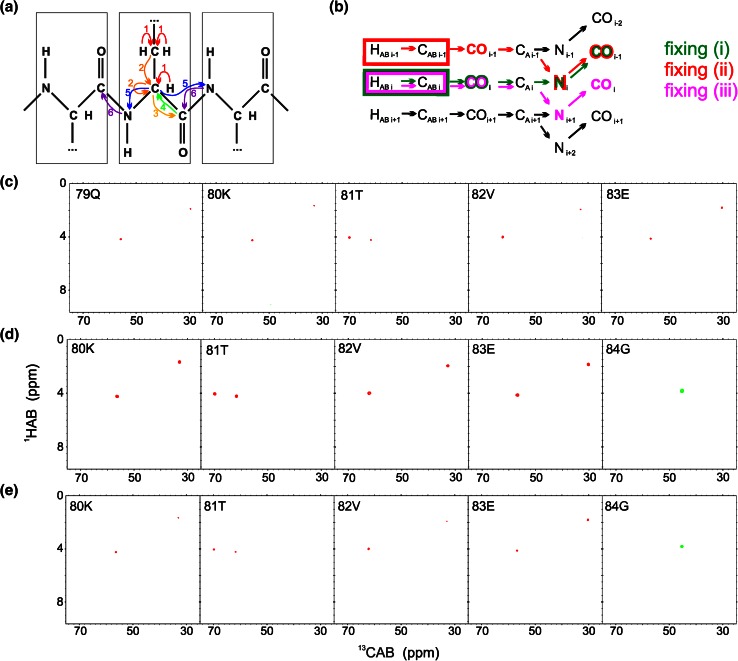
5D HabCabCO(CA)NCO technique. **a** Coherence transfer in a polypeptide chain. **b** Three ways of fixing the frequencies for SMFT processing, shown using *different colors* on a coherence transfer scheme: (*1i*)—*green*, (*1ii*)—*red*, (*1iii*)—*magenta* (different fixing *symbols* are explained in the text). Highlighting the text with an appropriate *color* shows the frequencies fixed during SMFT, the frequencies appearing on a cross-section are marked using frames of the *same color*. **c**–**e** 2D cross-sections of the 5D spectrum of alpha-synuclein protein, obtained by SMFT procedure, using (*1i*) fixing (**c**), (*1ii*) fixing (**d**), (*1iii*) fixing (**e**). The cross-sections correspond to the 80K–84G protein fragment. Cross-sections corresponding to the same basis peak are placed one over another

5D HabCabCO(CA)NCO pulse sequence is shown in Fig. S2 and magnetization transfer pathway is shown in Fig. 2a. The numbering of experimental dimensions (used below) is the following: Hab^1^Cab^2^CO^3^(CA)N^4^CO^5^ (dimension 5 is directly detected). The spectrum contains peaks of four types: HA_i_–CA_i_–CO_i_–N_i_–CO_i−1_ (positive intensity unless residue *i* is glycine), HB_i_–CB_i_–CO_i_–N_i_–CO_i−1_ (always positive intensity), HA_i_–CA_i_–CO_i_–N_i+1_–CO_i_ (positive intensity unless residue *i* is glycine), HB_i_–CB_i_–CO_i_–N_i+1_–CO_i_ (always positive intensity). Three versions of fixing are possible for this experiment (Fig. 2b): (2i) dimension 3 fixed based on CO_i_ frequencies, dimension 4 fixed based on N_i_ frequencies and dimension 5 fixed based on CO_i−1_ frequencies: on such a cross-section peaks of two types appear: HA_i_–CA_i_ and HB_i_–CB_i_, (2ii) dimensions 3 and 5 fixed based on CO_i−1_ frequencies and dimension 4 fixed based on N_i_ frequencies: on such a cross-section peaks of two types appear: HA_i−1_–CA_i−1_ and HB_i−1_–CB_i−1_, (2iii) dimensions 3 and 5 fixed based on CO_i_ frequencies and dimension 4 fixed based on N_i+1_ frequencies: it yields identical peak types as version (2i), thus it can be used for cross-check in a case of overlapping planes. The overall results of this experiment are two consecutive HA–CA and HB–CB pairs, found on cross-sections corresponding to a single basis peak. Sample 2D cross-sections obtained from 5D HabCabCO(CA)NCO experiment are shown in Fig. 2c–e.

**Fig. 3:**
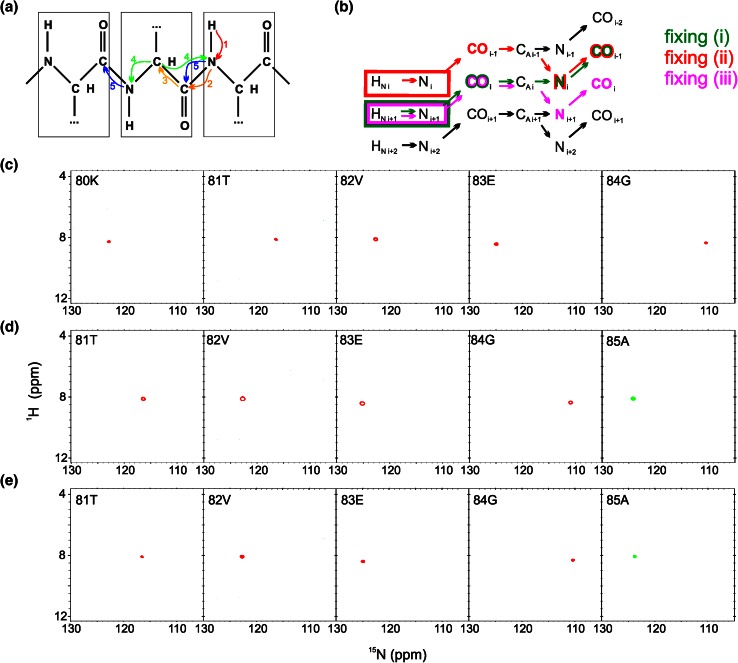
5D HNCO(CA)NCO technique. **a** Coherence transfer in a polypeptide chain. **b** Three ways of fixing the frequencies for SMFT processing, shown using different *colors* on a coherence transfer scheme: (*1i*)—*green*, (*1ii*)—*red*, (*1iii*)—*magenta* (different fixing *symbols* are explained in the text). Highlighting the text with an appropriate *color* shows the frequencies fixed during SMFT, the frequencies appearing on a cross-section are marked using frames of the *same color*. **c**–**e** 2D cross-sections of the 5D spectrum of alpha-synuclein protein, obtained by SMFT procedure, using (*1i*) fixing (**c**), (*1ii*) fixing (**d**), (*1iii*) fixing (**e**). The cross-sections correspond to the 80K–84G protein fragment. Cross-sections corresponding to the same basis peak are placed one over another

5D HNCO(CA)NCO pulse sequence is shown in Fig. S3 and magnetization transfer pathway is shown in Fig. 3a. The numbering of experimental dimensions (used below) is the following: H^N 1^N^2^CO^3^(CA)N^4^CO^5^ (dimension 5 is directly detected). The spectrum contains peaks of two types: H_i_^N^–N_i_–CO_i−1_–N_i−1_–CO_i−2_ (positive intensity unless residue *i* − 1 is glycine), H_i_^N^–N_i_–CO_i−1_–N_i_–CO_i−1_ (positive intensity unless residue *i* − 1 is glycine). Similarly to the 5D experiment above, also here three versions of fixing are possible (Fig. 3b): (3i) dimension 3 fixed based on CO_i_ frequencies, dimension 4 fixed based on N_i_ frequencies and dimension 5 fixed based on CO_i−1_ frequencies: on such a cross-section just a single peak appears: H_i+1_^N^–N_i+1_, (3ii) dimensions 3 and 5 fixed based on CO_i−1_ frequencies and dimension 4 fixed based on N_i_ frequencies: on such a cross-section a peak of the following type appears: H_i_^N^–N_i_, (3iii) dimensions 3 and 5 fixed based on CO_i_ frequencies and dimension 4 fixed based on N_i+1_ frequencies: it yields identical peak types as version (3i), thus it can be used for cross-check in a case of overlapping planes. In general, this experiment yields two consecutive H^N^–N pairs, found on cross-sections corresponding to a single basis peak. Sample 2D cross-sections obtained from 5D HNCO(CA)NCO experiment are shown in Fig. 3c–e.

### The strategy of resonance assignment and multiple fixing method

The strategy of resonance assignment is based on the parallel analysis of cross-sections of various multidimensional spectra. The four-level process, performed automatically by the TSAR program (Zawadzka-Kazimierczuk et al. 2012a), includes: (1) formation of cross-section spin systems (CSSSs, a data structure corresponding to each basis peak, containing the chemical shifts of the nuclei of the i − 2, i − 1, i, i + 1 and i + 2 residues), (2) finding the sequential connectivities between the CSSSs and formation of CSSSs chains, (3) recognition of amino acids that may correspond to each CSSS, (4) mapping of the CSSSs chains on the protein sequence.

**Fig. 4:**
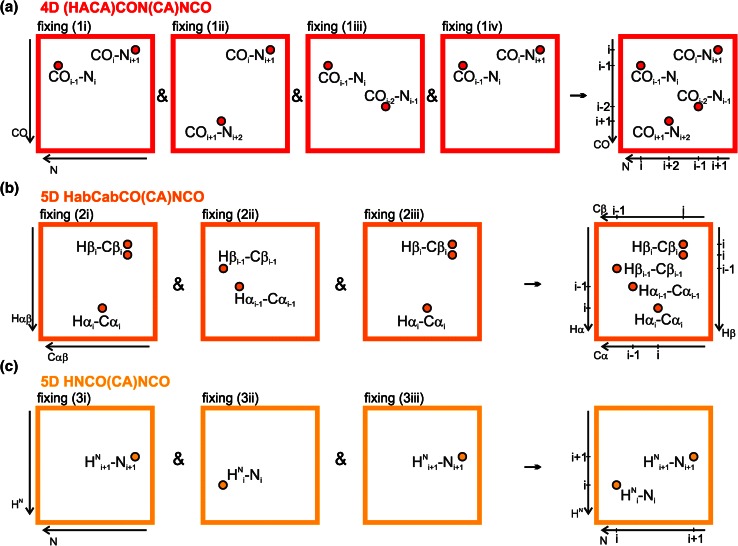
Schemes of cross-sections corresponding to a *single basis peak* CO_i_–N_i+1_–N_i_–CO_i−1_. For all the techniques, cross-section of each way of fixing is shown. On a *right*-*hand side* of each *panel*, a hypothetical plane containing peaks from all types of fixing is shown (in practice there is no such a cross-section)

Multiple fixing significantly facilitates steps (1) and (4) of the procedure. The cross-sections originating from various spectra and various ways of fixing are all calculated using the same basis peak list. Therefore, there are several 2D planes corresponding to each basis peak. These planes together contain peaks of various types and allow extending the range of chemical shifts accessible in each CSSS. For instance, the 4D (HACA)CON(CA)NCO spectrum when processed using a single fixing yields information on two consecutive CO–N pairs. When the same spectrum is processed using additionally two other fixing methods four consecutive CO–N pairs are included into the CSSS. It will give the establishment of the sequential links via three CO–N pairs instead of just one. This makes the technique applicable to the complex cases with high degree of CO–N overlap. Another example is 5D HNCO(CA)NCO spectrum, which fixed in one way yields just one H^N^–N pair, not allowing to establish any sequential links. Other fixing provides the second H^N^–N pair, making the sequential linking possible. In total, all the proposed experiments, processed using multiple fixing SMFT, yield the following chemical shifts: CO_i−2_, CO_i−1_, CO_i_, CO_i+1_, N_i−1_, N_i_, N_i+1_, N_i+2_, CA_i−1_, CA_i_, CB_i−1_, CB_i_, HA_i−1_, HA_i_, HB_i−1_, HB_i_, H_i_^N^, H_i+1_^N^ (see Fig. 4).

**Fig. 5:**
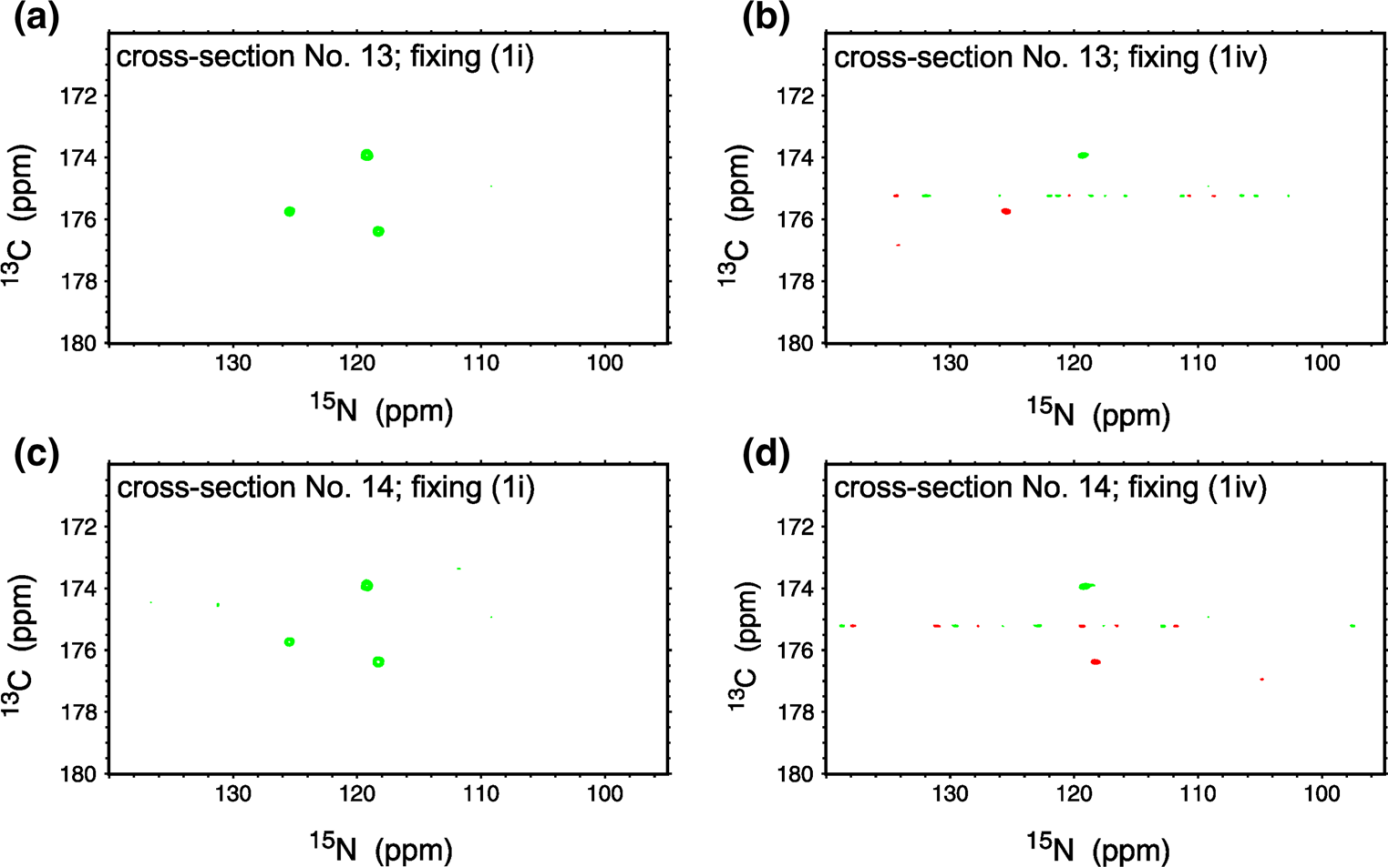
An example of 4D (HACA)CON(CA)NCO (*1i*) cross-sections overlap: three peaks instead of the expected two appear on both cross-sections (number *13* and *14*). The ambiguity can be resolved by comparison with the same cross-sections calculated using (*1iv*) fixing, where identical peaks are expected

Multiple fixing might help also in the case of cross-section overlap, when two different ways of fixing result in identical set of peaks. This is the case e.g. in 4D (HACA)CON(CA)NCO spectrum. Peaks CO_i−1_–N_i_ and CO_i_–N_i+1_ appear on two cross-sections, which are calculated by fixing N_i_ and CO_i−1_ or N_i+1_ and CO_i_ frequencies. It may happen that N_i_–CO_i−1_ pair has chemical shifts close to another N_j_–CO_j−1_, which results in excess of peaks on both overlapping planes. It may also happen that N_i+1_–CO_i_ pair is close to another N_k_–CO_k−1_, and also in these two planes there are more peaks than expected. However most likely the “additional” peaks in N_i_–CO_i−1_ and N_i+1_–CO_i_ planes are not identical. This difference can be used to resolve the overlap (see Fig. 5) and again confirms the high functionality of the proposed techniques for IDPs with the large extent of N–CO overlap.

It is worth mentioning that not all ways of fixing are equally useful. On those where direct dimension is not fixed the ridges of artifacts along the indirect dimension may appear, making such planes more difficult to work with than those with fixed direct dimension. The contribution of sampling artifacts to the overall spectral noise depends on the technique sensitivity. In a case of low-sensitive experiments with the low dynamic range of peak amplitudes, including typically all ^13^C-detected ones, the artifacts constitute just a tiny proportion of all spectral noise. For more sensitive techniques, the problem of spectral artifacts can be overcome by using one of the artifact-cleaning methods (Kazimierczuk et al. 2007; Coggins and Zhou 2008; Stanek and Koźmiński 2010). Various cross-section types may differ in a degree of planes overlap. It depends on the resolution in fixed dimensions. As direct dimension features the highest resolution, usually the planes obtained without direct dimension fixing overlap more severely than those calculated with direct-dimension fixing. Usually, if two types of fixing yield identical peaks, it is more beneficial to use the one with direct-dimension fixing. In the proposed 5D experiments (HabCabCO(CA)NCO and HNCO(CA)NCO) in one fixing type (2i and 3i) three different frequencies are used for cross-sections calculation, while in two other fixing types (2ii and 3ii, 2iii and 3iii) just two different frequencies are used—dimensions 3 and 5 are fixed based on the same frequency (see Figs. 2b, 3b). Therefore, sets of frequencies used for fixing 2i and 3i are more unique then those used for other fixing types. It results with lower degree of cross-section overlap for fixing 2i and 3i.

In principle, a full-dimensional spectrum contains all the information available from the given experiment. Obviously, multiple fixing will not yield any new information, but provides an easy access to the available information. It facilitates expanding of the CSSSs to promote finding more sequential connectivities. In consequence the assignment process is more efficient.

The difficulties in the assignment process may appear when some peaks are missing in the basis spectrum. It can happen due to the low sensitivity of the basis experiment or due to overlap of the CO_i_–N_i+1_–N_i_–CO_i−1_ and CO_i_–N_i+1_–N_i+1_–CO_i_ peaks, which have opposite signs. Then, the whole CSSS is missing, as the corresponding cross-sections of the high-dimensional spectra are not calculated. This may interrupt the formation of chains of CSSS. Nonetheless, as the basis spectrum yields the sequential connectivities via three N–CO pairs, such a gap is not critical. Shorter chains (with a gap between) can be still sequentially linked and all the chemical shifts of the “lacking” residue can be acquired from the adjacent CSSSs.

Due to high dimensionality of the experiments, the strategy is capable to cope with proteins with high degree of chemical shift overlap. Excitation of aliphatic proton nuclei (utilized in two of the three spectra used) and ^13^C-detection makes it better suited to proline-rich IDPs than more sensitive, in the case of slow chemical exchange, H^N^-excited and/or—detected techniques. Comparing to previously published strategies employing ^13^C-detected high-dimensional experiments (Nováček et al. 2011, 2012, 2013; Bermel et al. 2012, 2013b), the proposed strategy seems to be very robust, as the sequential connectivities are established via chemical shifts of seven nuclei types: N, CO, H^N^, CA, CB, HA and HB. The former three ones (N, CO, HN) are especially useful in case of IDPs. They are typically better resolved than the aliphatic carbon and proton chemical shifts, which in IDPs are strongly dependent on the particular amino acid. Moreover, two of the chemical shifts (N and CO) provide the links between four residues. Similar level of sequential linking was proposed in (Bermel et al. 2013c), which can use (dependently on the set of chosen pulse sequences) N and CO chemical shifts of three residues and H_N_, CA, CB, HA and HB of two residues. The main difference between the two strategies are the fixed dimensions, which influence the level of cross-section overlap. In the strategy of Bermel et al. in 4D pulse sequences two frequencies (CO_i−1_ and N_i_) are fixed for SMFT and in 5D pulse sequences CA_i−1_ is fixed additionally. In the strategy proposed in the present article, in one fixing type of the 5D techniques, CO_i_, N_i_ and CO_i−1_ frequencies are fixed. Such triples are better resolved than the CA_i−1_, CO_i−1_ and N_i_ triples utilized by Bermel et al., which is beneficial, regarding the level of cross-section overlap. On the other hand, other fixing types of the 5D spectra and the basis spectrum utilize CO_i−1_ and N_i_ pairs, which not always allows to resolve the cross-sections. To sum up, the two approaches are of similar quality.

### Experimental results

All presented techniques were tested on alpha-synuclein protein and the data were used to assign the resonances. To get the assignment at least one experiment assuring the sequential connectivities and one experiment yielding CB chemical shifts (for amino acid recognition) are needed. Thus, assignment can be done using just the 4D (HACA)CON(CA)NCO and 5D HabCabCO(CA)NCO data (below we call it data set 1). However, such a data set does not yield H^N^ chemical shifts. Therefore, also the second data set (data set 2) was constructed, including additionally 5D HNCO(CA)NCO spectrum. Data set 1 was acquired within ca. 132 h of the measurement time, while data set 2—within ca. 213 h. Due to the high signal to noise ratio in the 5D spectra, the data was transformed again using smaller number of increments (on NUS data such operation does not cause resolution loss). It was found that both 5D HabCabCO(CA)NCO and 5D HNCO(CA)NCO spectra obtained using 1200 increments (2/3 of the original number of increments) contained identical set of peaks: no false peak appeared and no true peak disappeared. It means that these experiments could be acquired faster, without any information loss. In such a case, the total measurement time would be <106 h for data set 1, and almost 160 h for data set 2.

The results obtained by the TSAR program are gathered in Table 2. They are satisfactory in both cases. For data set 1 the fraction of correctly and incorrectly assigned resonances was 91.8 and 1.2 % (respectively), while for data set 2 it was 92.9 and 1.2 % (respectively). It might seem surprising that the highest percentage of incorrect assignments corresponds to CO and N, which are the nuclei that form the basis of the assignment method. To understand this fact, one should consider also the fractions of correctly assigned resonances, which are also the highest for CO and N nuclei. Simply, more spectral peaks originating from aliphatic groups than from amide and carbonyl groups were missing. Also, in some cases when one of the aliphatic peaks (HA–CA or HB–CB) was missing, then TSAR program could not decide whether the present peak was alpha or beta peak, and in consequence this peak remained unassigned. In the presented data, for some incorrectly assigned CO and N nuclei, no other chemical shift was known, thus the percentage of incorrect assignments for nuclei other than CO and N did not increase.

**Table 2:** Results obtained with the TSAR program

Data set	Total measurement (time, h)	Assigned resonances correct/incorrect (%)							
		H^N^	N	CO	CA	CB	HA	HB	total
1	132 (106)^a^	n.a.	96.4/1.4	96.4/2.9	90.7/0.7	87.7/0.8	90.7/0.7	87.7/0.8	91.8/1.2
2	213 (160)^a^	94.8/0.7	96.4/1.4	96.4/2.9	90.7/0.7	87.7/0.8	90.7/0.7	87.7/0.8	92.2/1.2

^a^In parenthesis the restricted experimental times were given (the experimental times that provide identical results as full-time experiments, see the main text)

For both data sets the chains of cross-sections were identical. 123 cross-sections were correctly assigned. Most of them (106) were parts of long (≥8 residues) chains, whose assignment is the most reliable. Just three of them were parts of short (1–2 residues) chains. Out of three incorrectly assigned cross-sections, two were in “chains” of length 1 and one was at the very end of a long chain.

## Conclusion

In the present study we proposed two new high-dimensional experiments 5D HabCabCO(CA)NCO and 5D HNCO(CA)NCO) and a 4D (HACA)CON(CA)NCO extension of the previously published 3D experiments, dedicated for resonance assignment of proteins featuring low chemical shift dispersion, like IDPs. Since all these experiments exploit ^13^C detection and two of them use HA and HB proton excitation, this set of experiments is suitable for samples of high content of proline residue or featuring high level of chemical exchange of amide protons. NUS allows to acquire these experiments in limited measurement time. Using sparse multidimensional Fourier transform together with multiple fixing method provides an easy access to the high-dimensional data and facilitates spectral analysis. The TSAR program can perform the assignment automatically. On the example of 140-amino-acid-long IDP it was shown that the proposed techniques enable almost complete automatic resonance assignment.

## Electronic supplementary material

4D (HACA)CON(CA)NCO, 5D HabCabCO(CA)NCO and 5D HNCO(CA)NCO pulse sequences. Input file for the TSAR program, with definitions of all the experiments. (PDF 874 kb)
