# Supplementary material for: 13C-detected NMR experiments for automatic resonance assignment of IDPs and multiple-fixing SMFT processing
Source: J Biomol NMR. 2015 Apr 23;62(2):179–90. doi: 10.1007/s10858-015-9932-9 (PMC4451475; doi:10.1007/s10858-015-9932-9)
Supplement: Supplementary file 1 — 4D (HACA)CON(CA)NCO, 5D HabCabCO(CA)NCO and 5D HNCO(CA)NCO pulse sequences. Input file for the TSAR program, with definitions of all the experiments. (PDF 874 kb) [file 10858_2015_9932_MOESM1_ESM.pdf]

„13C-detected NMR experiments for automatic resonance assignment of IDPs and multiple-fixing SMFT processing”

Paweł Dziekański [1,2], Katarzyna Grudziąż [1], Patrik Jarvoll [3], Wiktor Koźmiński [1], Anna Zawadzka-Kazimierczuk [1] ✉

[1] University of Warsaw, Faculty of Chemistry, Biological and Chemical Research Centre, Żwirki i Wigury 101, 02-089 Warsaw, Poland

[2] University of Warsaw, Faculty of Physics, Pasteura 5, 02-093 Warsaw, Poland

[3] Agilent Technologies, 10 Mead Road, OX5 1QU Yarnton, United Kingdom

✉ e-mail: anzaw@chem.uw.edu.pl

SUPPLEMENTARY MATERIAL

The pulse sequences

The pulse sequences of 4D (HACA)CON(CA)NCO, 5D HabCabCON(CA)NCO and 5D HNCO(CA)NCO techniques are shown and the coherence transfer for each pulse sequence is described.

The rectangles stand for hard (non-selective) pulses, the round shapes stand for ^{13}C band-selective pulses. Filled and empty shapes correspond to 90 degree and 180 degree pulses, respectively. The train of empty rectangles corresponds to the six-element composite pulse affecting both CA and CO frequencies shaka6 (Shaka AJ, Chem Phys Lett 120 (1985) 201–205). The pulses were applied along the x axis unless noted differently. The color lines indicate the ^{13}C and ^{1}H carrier frequency, according to the following code: red - CO, green - Cα, blue - frequency between CO and Cα, purple - Cαβ, magenta - H^N, yellow - Hαβ. The line denoted with "gradient" corresponds to pulsed field gradients applied along the z-axis. In each technique the four-step phase cycle was used: ϕ_1 = x, -x, ϕ_2 = 2x, 2(-x) and $\phi_{rec} = \phi_1 + \phi_2$ (the ϕ_1 and ϕ_2 symbols are shown in the pictures). For the acquisition of IPAP components the following values of delays a, b, c, d and phase ψ (shown in the pictures) were used: IP: $a_{IP} = b_{IP} = c_{IP} = d_{IP}$ = 7 ms, ψ = x; AP: a_{AP} = 4.5 ms, b_{AP} =9.5 ms, c_{AP} = 14.0 ms, d_{AP}= 0.0 ms, ψ = -x.

The orange dashed lines (named with capital letters) show the moments of each pulse sequence for which product operators and appropriate sensitivity factors are given below. To express the sensitivity factors, the following notation was used:

- values of scalar coupling constants:

$J_{HA\text{-}CA}$, $J_{HB\text{-}CB}$, $J_{CA\text{-}CO}$, $J_{CA\text{-}CB}$, $J_{N\text{-}CO}$, $^{1}J_{N\text{-}CA}$ (for one-bond N-CA coupling constant), $^{2}J_{N\text{-}CA}$ (for two-bond N-CA coupling constant), $J_{HN\text{-}N}$

- values of transverse relaxation times (longitudinal relaxation is neglected):

τ_{HA}, τ_{HB}, τ_{CA}, τ_{CB}, τ_{CO}, τ_{N}, τ_{HN}

- numbers of various nuclei in the residue:

k – number of HA proton nuclei in the residue
l – number of HB proton nuclei in the residue
m – number of CB carbon nuclei in the residue
n – number of CG carbon nuclei in the residue

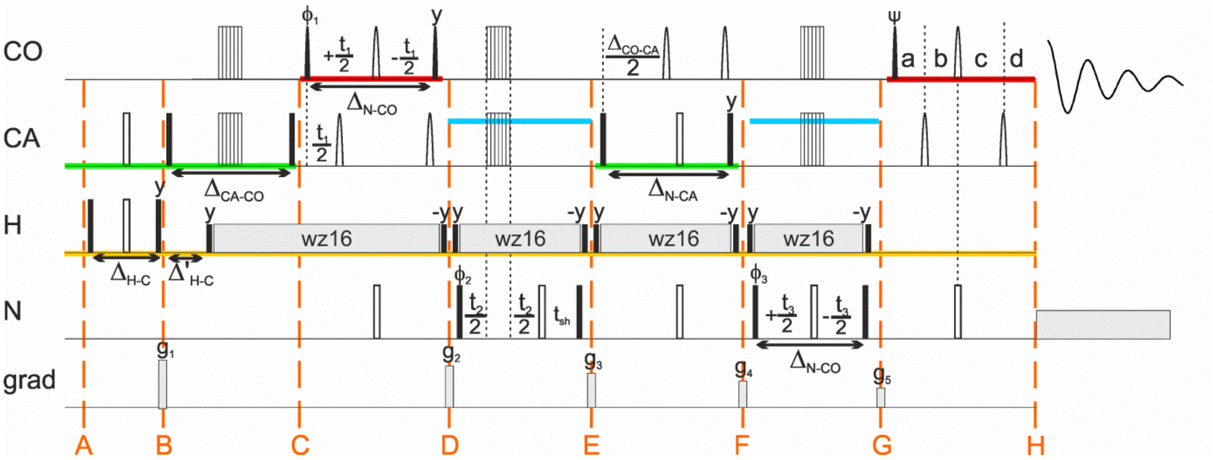

Figure S1. The pulse sequence of 4D (HACA)CON(CA)NCO technique. Evolution of second indirect dimension (N) is in real-time mode. Evolutions of first and third indirect dimensions (CO and N) are in constant-time mode: $+t_i/2$ or $-t_i/2$ in the picture mean adding or subtracting these values from half of the appropriate Δ delay. The delays were set as follows: $\Delta_{H\text{-}C}$ = 3.7 ms, $\Delta'_{H\text{-}C}$ = 2.2 ms, $\Delta_{CA\text{-}CO}$ = 6.3 ms, $\Delta_{N\text{-}CO}$ = 28.0 ms, $\Delta_{CO\text{-}CA}$ = 9.1 ms, $\Delta_{N\text{-}CA}$ = 28.6 ms and t_{sh} was set to the length of the composite shaka6 pulse. Quadrature detection in the indirect dimensions was achieved by incrementing phases ϕ_1, ϕ_2 and ϕ_3 in a States-TPPI manner. The gradient intensities were set as follows: g_1 = 0.206 T/m, g_2 = 0.150 T/m, g_3 = 0.094 T/m, g_4 = 0.090 T/m, g_5 = 0.056 T/m.

Coherence transfer for 4D (HACA)CON(CA)NCO pulse sequence:

A: $\mathbf{HA_z^i}$

B: $\mathbf{2\,HA_z^i\,CA_z^i} \cdot SF_B$

$SF_B = \sin(\pi \cdot J_{HA\text{-}CA} \cdot \Delta_{H\text{-}C}) \cdot \exp(-\Delta_{H\text{-}C} / \tau_{HA})$

C: $\mathbf{2\,CA_z^i\,CO_z^i} \cdot SF_B \cdot SF_C$

$SF_C = \sin(\pi \cdot J_{HA\text{-}CA} \cdot \Delta'_{H\text{-}C}) \cdot \cos^{k-1}(\pi \cdot J_{HA\text{-}CA} \cdot \Delta'_{H\text{-}C}) \cdot \sin(\pi \cdot J_{CA\text{-}CO} \cdot \Delta_{CA\text{-}CO}) \cdot \cos^{m}(\pi \cdot J_{CA\text{-}CB} \cdot \Delta_{CA\text{-}CO}) \cdot \exp(-\Delta_{CA\text{-}CO} / \tau_{CA})$

D: $\mathbf{4\,CA_z^i\,CO_z^i\,N_z^{i+1}} \cdot SF_B \cdot SF_C \cdot SF_D$

$SF_D = \sin(\pi \cdot J_{N\text{-}CO} \cdot \Delta_{N\text{-}CO}) \cdot \exp(-\Delta_{N\text{-}CO} / \tau_{CO})$

E: $\mathbf{4\,CA_z^i\,CO_z^i\,N_z^{i+1}} \cdot SF_B \cdot SF_C \cdot SF_D \cdot SF_E$

$SF_E = \exp(-t_2 / \tau_N)$ (t_{sh}, during which the relaxation of N nuclei takes place as well, is time of about 144 μs, which is neglible compared with typical evolution delays, which are at the order of tens of ms)

F: $\mathbf{2\,CA_z^i\,N_z^{i+1}} \cdot SF_B \cdot SF_C \cdot SF_D \cdot SF_E \cdot SF_{F1} + \mathbf{2\,CA_z^i\,N_z^i} \cdot SF_B \cdot SF_C \cdot SF_D \cdot SF_E \cdot SF_{F2}$

$SF_{F1} = \sin(\pi \cdot J_{CA\text{-}CO} \cdot \Delta_{CO\text{-}CA}) \cdot \cos(\pi \cdot {}^1J_{N\text{-}CA} \cdot \Delta_{N\text{-}CA}) \cdot \cos(\pi \cdot {}^2J_{N\text{-}CA} \cdot \Delta_{N\text{-}CA}) \cdot \cos^{m}(\pi \cdot J_{CA\text{-}CB} \cdot \Delta_{N\text{-}CA}) \cdot \exp(-\Delta_{N\text{-}CA}/\tau_{CA})$

$SF_{F2} = \sin(\pi \cdot J_{CA\text{-}CO} \cdot \Delta_{CO\text{-}CA}) \cdot \sin(\pi \cdot {}^1J_{N\text{-}CA} \cdot \Delta_{N\text{-}CA}) \cdot \sin(\pi \cdot {}^2J_{N\text{-}CA} \cdot \Delta_{N\text{-}CA}) \cdot \cos^{m}(\pi \cdot J_{CA\text{-}CB} \cdot \Delta_{N\text{-}CA}) \cdot \exp(-\Delta_{N\text{-}CA}/\tau_{CA})$

G: $\mathbf{2\ N_z^{i+1}\ CO_z^{i}} \cdot SF_B \cdot SF_C \cdot SF_D \cdot SF_E \cdot SF_{F1} \cdot SF_{G1} + \mathbf{2\ N_z^{i}\ CO_z^{i-1}} \cdot SF_B \cdot SF_C \cdot SF_D \cdot SF_E \cdot SF_{F2} \cdot SF_{G2}$

$$SF_{G1} = \sin(\pi \cdot J_{N\text{-}CO} \cdot \Delta_{N\text{-}CO}) \cdot \sin(\pi \cdot {}^2J_{N\text{-}CA} \cdot \Delta_{N\text{-}CO}) \cdot \cos(\pi \cdot {}^1J_{N\text{-}CA} \cdot \Delta_{N\text{-}CO}) \cdot \exp(-\Delta_{N\text{-}CO} / \tau_N)$$

$$SF_{G2} = \sin(\pi \cdot J_{N\text{-}CO} \cdot \Delta_{N\text{-}CO}) \cdot \sin(\pi \cdot {}^1J_{N\text{-}CA} \cdot \Delta_{N\text{-}CO}) \cdot \cos(\pi \cdot {}^2J_{N\text{-}CA} \cdot \Delta_{N\text{-}CO}) \cdot \exp(-\Delta_{N\text{-}CO} / \tau_N)$$

H in-phase component:

$\mathbf{CO_x^{i}} \cdot SF_B \cdot SF_C \cdot SF_D \cdot SF_E \cdot SF_{F1} \cdot SF_{G1} \cdot SF_{H1} + \mathbf{CO_x^{i-1}} \cdot SF_B \cdot SF_C \cdot SF_D \cdot SF_E \cdot SF_{F2} \cdot SF_{G2} \cdot SF_{H1}$

$$SF_{H1} = \sin(\pi \cdot J_{N\text{-}CO} \cdot (a_{IP}+b_{IP}+c_{IP}+d_{IP})) \cdot \exp(-(a_{IP}+b_{IP}+c_{IP}+d_{IP}) / \tau_{CO})$$

H anti-phase component:

$\mathbf{2\ CO_y^{i}\ CA_z^{i}} \cdot SF_B \cdot SF_C \cdot SF_D \cdot SF_E \cdot SF_{F1} \cdot SF_{G1} \cdot SF_{H2} + \mathbf{2\ CO_y^{i-1}\ CA_z^{i-1}} \cdot SF_B \cdot SF_C \cdot SF_D \cdot SF_E \cdot SF_{F2} \cdot SF_{G2} \cdot SF_{H2}$

$$SF_{H2} = \sin(\pi \cdot J_{N\text{-}CO} \cdot (a_{AP}+b_{AP}+c_{AP}+d_{AP})) \cdot \sin(\pi \cdot J_{CA\text{-}CO} \cdot (a_{AP}-b_{AP}+c_{AP}-d_{AP})) \cdot \exp(-(a_{AP}+b_{AP}+c_{AP}+d_{AP}) / \tau_{CO})$$

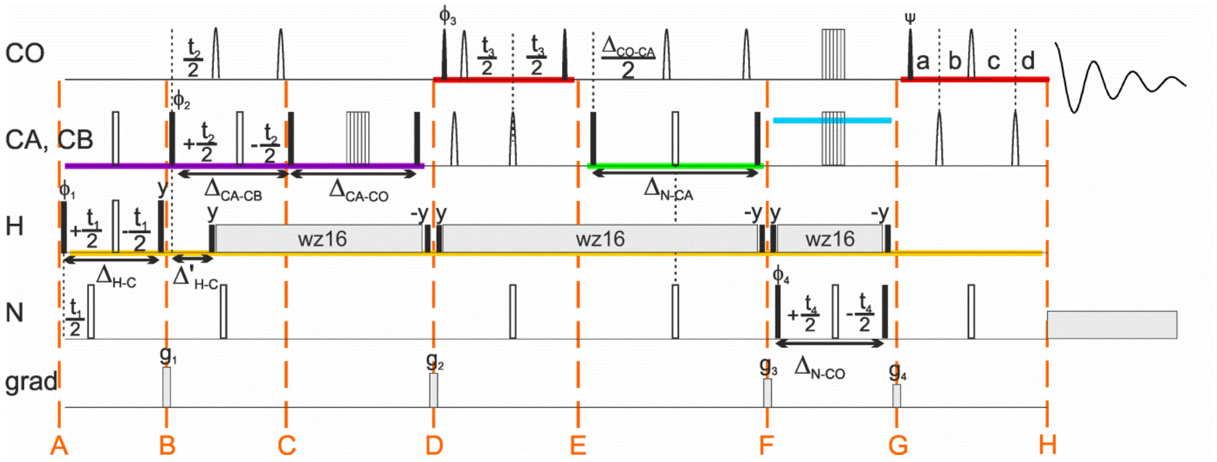

Figure S2. The pulse sequence of 5D HabCabCO(CA)NCO technique. Evolution of third indirect dimension (CO) is in real-time mode. Evolutions of first, second and fourth indirect dimensions (Hab, Cab and N) are in constant-time mode: $+t_i/2$ or $-t_i/2$ in the picture mean adding or subtracting these values from half of the appropriate Δ delay. The delays were set as follows: $\Delta_{H\text{-}C}$ = 3.7 ms, $\Delta'_{H\text{-}C}$ = 2.2 ms, $\Delta_{CA\text{-}CB}$ = 14.3 ms, $\Delta_{CA\text{-}CO}$ = 6.8 ms, $\Delta_{CO\text{-}CA}$ = 9.1 ms, $\Delta_{N\text{-}CA}$ = 28.0 ms and $\Delta_{N\text{-}CO}$ = 28.0 ms. Quadrature detection in the indirect dimensions was achieved by incrementing phases ϕ_1, ϕ_2, ϕ_3 and ϕ_4 in a States-TPPI manner. The gradient intensities were set as follows: g_1 = 0.206 T/m, g_2 = 0.150 T/m, g_3 = 0.094 T/m, g_4 = 0.090 T/m.

Coherence transfer for 5D HabCabCO(CA)NCO pulse sequence:

A: $\mathbf{HA_z^{i} + HB_z^{i}}$

B: $\mathbf{2\ HA_z^{i}\ CA_z^{i}} \cdot SF_{B1} + \mathbf{2\ HB_z^{i}\ CB_z^{i}} \cdot SF_{B2}$

$$SF_{B1} = \sin(\pi \cdot J_{HA\text{-}CA} \cdot \Delta_{H\text{-}C}) \cdot \exp(-\Delta_{H\text{-}C} / \tau_{HA})$$

$$SF_{B2} = \sin(\pi \cdot J_{HB\text{-}CB} \cdot \Delta_{H\text{-}C}) \cdot \exp(-\Delta_{H\text{-}C} / \tau_{HB})$$

C: $\mathbf{CA_x^i} \cdot SF_{B1} \cdot SF_{C1}$ + $\mathbf{2\ CB_y^i\ CA_z^i} \cdot SF_{B2} \cdot SF_{C2}$

$$SF_{C1} = \sin(\pi \cdot J_{HA\text{-}CA} \cdot \Delta'_{H\text{-}C}) \cdot \cos^{k-1}(\pi \cdot J_{HA\text{-}CA} \cdot \Delta'_{H\text{-}C}) \cdot \cos^{m}(\pi \cdot J_{CA\text{-}CB} \cdot \Delta_{CA\text{-}CB}) \cdot \exp(-\Delta_{CA\text{-}CB} / \tau_{CA})$$

$$SF_{C2} = \sin(\pi \cdot J_{HB\text{-}CB} \cdot \Delta'_{H\text{-}C}) \cdot \cos^{l-1}(\pi \cdot J_{HB\text{-}CB} \cdot \Delta'_{H\text{-}C}) \cdot \sin(\pi \cdot J_{CA\text{-}CB} \cdot \Delta_{CA\text{-}CB}) \cdot \cos^{n}(\pi \cdot J_{CB\text{-}CG} \cdot \Delta_{CA\text{-}CB}) \cdot \exp(-\Delta_{CA\text{-}CB} / \tau_{CB})$$

D: $\mathbf{2\ CA_z^i\ CO_z^i} \cdot SF_{B1} \cdot SF_{C1} \cdot SF_{D1}$ + $\mathbf{2\ CA_z^i\ CO_z^i} \cdot SF_{B2} \cdot SF_{C2} \cdot SF_{D2}$ (one of the apparently identical components is modulated with oscillatory functions of the HA and CA frequencies, while the other – of the HB and CB frequencies)

$$SF_{D1} = \cos(\pi \cdot J_{CA\text{-}CB} \cdot \Delta_{CA\text{-}CO}) \cdot \sin(\pi \cdot J_{CA\text{-}CO} \cdot \Delta_{CA\text{-}CO}) \cdot \exp(-\Delta_{CA\text{-}CO} / \tau_{CA})$$

$$SF_{D2} = \sin(\pi \cdot J_{CA\text{-}CB} \cdot \Delta_{CA\text{-}CO}) \cdot \sin(\pi \cdot J_{CA\text{-}CO} \cdot \Delta_{CA\text{-}CO}) \cdot \exp(-\Delta_{CA\text{-}CO} / \tau_{CA})$$

E: $\mathbf{2\ CA_z^i\ CO_z^i} \cdot SF_{B1} \cdot SF_{C1} \cdot SF_{D1} \cdot SF_E$ + $\mathbf{2\ CA_z^i\ CO_z^i} \cdot SF_{B2} \cdot SF_{C2} \cdot SF_{D2} \cdot SF_E$

$$SF_E = \exp(-t_3 / \tau_N)$$

F: $\mathbf{2\ CA_z^i\ N_z^i} \cdot SF_{B1} \cdot SF_{C1} \cdot SF_{D1} \cdot SF_E \cdot SF_{F1}$ + $\mathbf{2\ CA_z^i\ N_z^i} \cdot SF_{B2} \cdot SF_{C2} \cdot SF_{D2} \cdot SF_E \cdot SF_{F1}$

+ $\mathbf{2\ CA_z^i\ N_z^{i+1}} \cdot SF_{B1} \cdot SF_{C1} \cdot SF_{D1} \cdot SF_E \cdot SF_{F2}$ + $\mathbf{2\ CA_z^i\ N_z^{i+1}} \cdot SF_{B2} \cdot SF_{C2} \cdot SF_{D2} \cdot SF_E \cdot SF_{F2}$

$$SF_{F1} = \sin(\pi \cdot J_{CA\text{-}CO} \cdot \Delta_{CO\text{-}CA}) \cdot \sin(\pi \cdot {}^1J_{N\text{-}CA} \cdot \Delta_{N\text{-}CA}) \cdot \cos(\pi \cdot {}^2J_{N\text{-}CA} \cdot \Delta_{N\text{-}CA}) \cdot \cos^{m}(\pi \cdot J_{CA\text{-}CB} \cdot \Delta_{N\text{-}CA}) \cdot \exp(-\Delta_{N\text{-}CA}/\tau_{CA})$$

$$SF_{F2} = \sin(\pi \cdot J_{CA\text{-}CO} \cdot \Delta_{CO\text{-}CA}) \cdot \sin(\pi \cdot {}^2J_{N\text{-}CA} \cdot \Delta_{N\text{-}CA}) \cdot \cos(\pi \cdot {}^1J_{N\text{-}CA} \cdot \Delta_{N\text{-}CA}) \cdot \cos^{m}(\pi \cdot J_{CA\text{-}CB} \cdot \Delta_{N\text{-}CA}) \cdot \exp(-\Delta_{N\text{-}CA}/\tau_{CA})$$

G: $\mathbf{2\ N_z^i\ CO_z^{i\text{-}1}} \cdot SF_{B1} \cdot SF_{C1} \cdot SF_{D1} \cdot SF_E \cdot SF_{F1} \cdot SF_{G1}$ + $\mathbf{2\ N_z^i\ CO_z^{i\text{-}1}} \cdot SF_{B2} \cdot SF_{C2} \cdot SF_{D2} \cdot SF_E \cdot SF_{F1} \cdot SF_{G1}$

+ $\mathbf{2\ N_z^{i+1}\ CO_z^i} \cdot SF_{B1} \cdot SF_{C1} \cdot SF_{D1} \cdot SF_E \cdot SF_{F2} \cdot SF_{G2}$ + $\mathbf{2\ N_z^{i+1}\ CO_z^i} \cdot SF_{B2} \cdot SF_{C2} \cdot SF_{D2} \cdot SF_E \cdot SF_{F2} \cdot SF_{G2}$

$$SF_{G1} = \sin(\pi \cdot J_{N\text{-}CO} \cdot \Delta_{N\text{-}CO}) \cdot \sin(\pi \cdot {}^1J_{N\text{-}CA} \cdot \Delta_{N\text{-}CO}) \cdot \cos(\pi \cdot {}^2J_{N\text{-}CA} \cdot \Delta_{N\text{-}CO}) \cdot \exp(-\Delta_{N\text{-}CO} / \tau_N)$$

$$SF_{G2} = \sin(\pi \cdot J_{N\text{-}CO} \cdot \Delta_{N\text{-}CO}) \cdot \sin(\pi \cdot {}^2J_{N\text{-}CA} \cdot \Delta_{N\text{-}CO}) \cdot \cos(\pi \cdot {}^1J_{N\text{-}CA} \cdot \Delta_{N\text{-}CO}) \cdot \exp(-\Delta_{N\text{-}CO} / \tau_N)$$

H in-phase component:

$\mathbf{CO_x^{i\text{-}1}} \cdot SF_{B1} \cdot SF_{C1} \cdot SF_{D1} \cdot SF_E \cdot SF_{F1} \cdot SF_{G1} \cdot SF_{H1}$ + $\mathbf{CO_x^{i\text{-}1}} \cdot SF_{B2} \cdot SF_{C2} \cdot SF_{D2} \cdot SF_E \cdot SF_{F1} \cdot SF_{G1} \cdot SF_{H1}$

+ $\mathbf{CO_x^i} \cdot SF_{B1} \cdot SF_{C1} \cdot SF_{D1} \cdot SF_E \cdot SF_{F2} \cdot SF_{G2} \cdot SF_{H1}$ + $\mathbf{CO_x^i} \cdot SF_{B2} \cdot SF_{C2} \cdot SF_{D2} \cdot SF_E \cdot SF_{F2} \cdot SF_{G2} \cdot SF_{H1}$

$$SF_{H1} = \sin(\pi \cdot J_{N\text{-}CO} \cdot (a_{IP}+b_{IP}+c_{IP}+d_{IP})) \cdot \exp(-(a_{IP}+b_{IP}+c_{IP}+d_{IP}) / \tau_{CO})$$

H anti-phase component:

$\mathbf{2\ CO_y^{i\text{-}1}\ CA_z^{i\text{-}1}} \cdot SF_{B1} \cdot SF_{C1} \cdot SF_{D1} \cdot SF_E \cdot SF_{F1} \cdot SF_{G1} \cdot SF_{H2}$ + $\mathbf{2\ CO_y^{i\text{-}1}\ CA_z^{i\text{-}1}} \cdot SF_{B2} \cdot SF_{C2} \cdot SF_{D2} \cdot SF_E \cdot SF_{F1} \cdot SF_{G1} \cdot SF_{H2}$

+ $\mathbf{2\ CO_y^i\ CA_z^i} \cdot SF_{B1} \cdot SF_{C1} \cdot SF_{D1} \cdot SF_E \cdot SF_{F2} \cdot SF_{G2} \cdot SF_{H2}$ + $\mathbf{2\ CO_y^i\ CA_z^i} \cdot SF_{B2} \cdot SF_{C2} \cdot SF_{D2} \cdot SF_E \cdot SF_{F2} \cdot SF_{G2} \cdot SF_{H2}$

$$SF_{H2} = \sin(\pi \cdot J_{N\text{-}CO} \cdot (a_{AP}+b_{AP}+c_{AP}+d_{AP})) \cdot \sin(\pi \cdot J_{CA\text{-}CO} \cdot (a_{AP}-b_{AP}+c_{AP}-d_{AP})) \cdot \exp(-(a_{AP}+b_{AP}+c_{AP}+d_{AP}) / \tau_{CO})$$

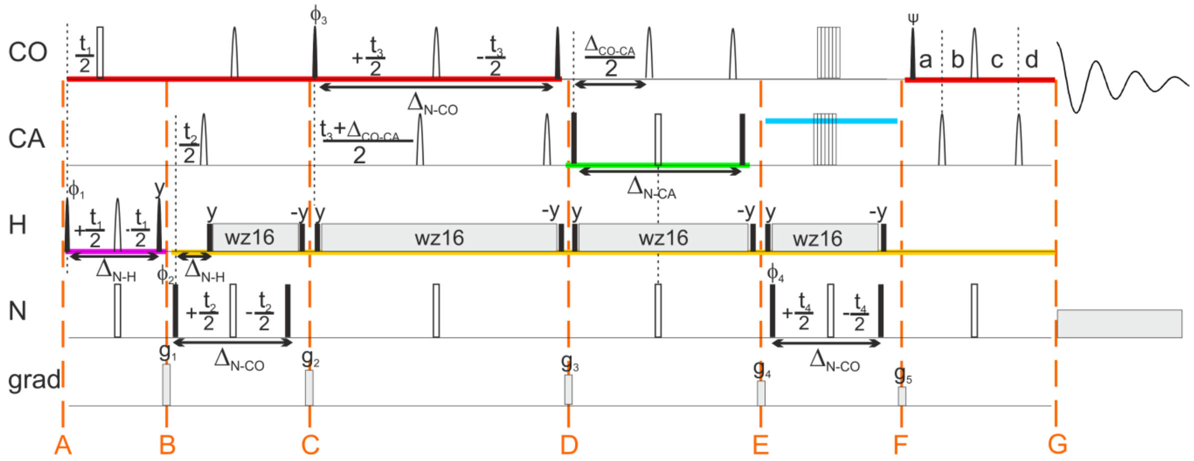

Figure S3. The pulse sequence of 5D HNCO(CA)NCO technique. Evolutions of all indirect dimensions (Hab, Cab, CO and N) are in constant-time mode: $+t_i/2$ or $-t_i/2$ in the picture mean adding or subtracting these values from half of the appropriate Δ delay. The delays were set as follows: $\Delta_{N\text{-}H}$ = 5.4 ms, $\Delta_{N\text{-}CO}$ = 28.0 ms, $\Delta_{CO\text{-}CA}$ = 9.1 ms and $\Delta_{N\text{-}CA}$ = 28.0 ms. Quadrature detection in the indirect dimensions was achieved by incrementing phases ϕ_1, ϕ_2, ϕ_3 and ϕ_4 in a States-TPPI manner. The gradient intensities were set as follows: g_1 = 0.206 T/m, g_2 = 0.150 T/m, g_3 = 0.094 T/m, g_4 = 0.090 T/m, g_5 = 0.056 T/m.

Coherence transfer for 5D HNCO(CA)NCO pulse sequence:

A: $\mathbf{HN_z^i}$

B: $\mathbf{2\,HN_z^i\,N_z^i} \cdot SF_B$

$$SF_B = \sin(\pi \cdot J_{HN\text{-}N} \cdot \Delta_{N\text{-}H}) \cdot \exp(-\Delta_{N\text{-}H} / \tau_{HN})$$

C: $\mathbf{2\,N_z^i\,CO_z^{i-1}} \cdot SF_B \cdot SF_C$

$$SF_C = \sin(\pi \cdot J_{HN\text{-}N} \cdot \Delta_{N\text{-}H}) \cdot \sin(\pi \cdot J_{N\text{-}CO} \cdot \Delta_{N\text{-}CO}) \cdot \exp(-\Delta_{N\text{-}CO} / \tau_N)$$

D: $\mathbf{2\,CO_z^{i-1}\,CA_z^{i-1}} \cdot SF_B \cdot SF_C \cdot SF_D$

$$SF_D = \sin(\pi \cdot J_{N\text{-}CO} \cdot \Delta_{N\text{-}CO}) \cdot \sin(\pi \cdot J_{CA\text{-}CO} \cdot \Delta_{CO\text{-}CA}) \cdot \exp(-\Delta_{N\text{-}CO} / \tau_{CO})$$

E: $\mathbf{2\,CA_z^{i-1}\,N_z^{i-1}} \cdot SF_B \cdot SF_C \cdot SF_D \cdot SF_{E1} + \mathbf{2\,CA_z^{i-1}\,N_z^i} \cdot SF_B \cdot SF_C \cdot SF_D \cdot SF_{E2}$

$$SF_{E1} = \sin(\pi \cdot J_{CA\text{-}CO} \cdot \Delta_{CO\text{-}CA}) \cdot \sin(\pi \cdot {}^1J_{N\text{-}CA} \cdot \Delta_{N\text{-}CA}) \cdot \cos(\pi \cdot {}^2J_{N\text{-}CA} \cdot \Delta_{N\text{-}CA}) \cdot \cos^m(\pi \cdot J_{CA\text{-}CB} \cdot \Delta_{N\text{-}CA}) \cdot \exp(-\Delta_{N\text{-}CA} / \tau_{CA})$$

$$SF_{E2} = \sin(\pi \cdot J_{CA\text{-}CO} \cdot \Delta_{CO\text{-}CA}) \cdot \sin(\pi \cdot {}^2J_{N\text{-}CA} \cdot \Delta_{N\text{-}CA}) \cdot \cos(\pi \cdot {}^1J_{N\text{-}CA} \cdot \Delta_{N\text{-}CA}) \cdot \cos^m(\pi \cdot J_{CA\text{-}CB} \cdot \Delta_{N\text{-}CA}) \cdot \exp(-\Delta_{N\text{-}CA} / \tau_{CA})$$

F: $\mathbf{2\,N_z^{i-1}\,CO_z^{i-2}} \cdot SF_B \cdot SF_C \cdot SF_D \cdot SF_{E1} \cdot SF_{F1} + \mathbf{2\,N_z^i\,CO_z^{i-1}} \cdot SF_B \cdot SF_C \cdot SF_D \cdot SF_{E2} \cdot SF_{F2}$

$$SF_{F1} = \sin(\pi \cdot J_{N\text{-}CO} \cdot \Delta_{N\text{-}CO}) \cdot \sin(\pi \cdot {}^1J_{N\text{-}CA} \cdot \Delta_{N\text{-}CO}) \cdot \cos(\pi \cdot {}^2J_{N\text{-}CA} \cdot \Delta_{N\text{-}CO}) \cdot \exp(-\Delta_{N\text{-}CO} / \tau_N)$$

$$SF_{F2} = \sin(\pi \cdot J_{N\text{-}CO} \cdot \Delta_{N\text{-}CO}) \cdot \sin(\pi \cdot {}^2J_{N\text{-}CA} \cdot \Delta_{N\text{-}CO}) \cdot \cos(\pi \cdot {}^1J_{N\text{-}CA} \cdot \Delta_{N\text{-}CO}) \cdot \exp(-\Delta_{N\text{-}CO} / \tau_N)$$

G in-phase component:

$$\mathbf{CO_x^{i-2}} \cdot SF_B \cdot SF_C \cdot SF_D \cdot SF_{E1} \cdot SF_{F1} \cdot SF_{G1} + \mathbf{CO_x^{i-1}} \cdot SF_B \cdot SF_C \cdot SF_D \cdot SF_{E2} \cdot SF_{F2} \cdot SF_{G1}$$

$$SF_{G1} = \sin(\pi \cdot J_{N\text{-}CO} \cdot (a_{IP}+b_{IP}+c_{IP}+d_{IP})) \cdot \exp(-(a_{IP}+b_{IP}+c_{IP}+d_{IP})/\tau_{CO})$$

G anti-phase component:

$$\mathbf{2\ CO_y^{i-2}\ CA_z^{i-2}} \cdot SF_B \cdot SF_C \cdot SF_D \cdot SF_{E1} \cdot SF_{F1} \cdot SF_{G2} + \mathbf{2\ CO_y^{i-1}\ CA_z^{i-1}} \cdot SF_B \cdot SF_C \cdot SF_D \cdot SF_{E2} \cdot SF_{F2} \cdot SF_{G2}$$

$$SF_{G2} = \sin(\pi \cdot J_{N\text{-}CO} \cdot (a_{AP}+b_{AP}+c_{AP}+d_{AP})) \cdot \sin(\pi \cdot J_{CA\text{-}CO} \cdot (a_{AP}-b_{AP}+c_{AP}-d_{AP})) \cdot \exp(-(a_{AP}+b_{AP}+c_{AP}+d_{AP})/\tau_{CO})$$

Input for the TSAR program

For using the TSAR program for automatic resonance assignment, all the experiments have to be properly defined, in a text file. The file starts with the definition of the basis experiment, after which the definitions of all other experiments follow. Importantly, each for a given experiment, each fixing type has to be defined separately (also the peak lists should be prepared from each fixing type separately). The syntax of the basis experiment definition is explained in Fig. S4. The syntax of other experiments definition is explained in Fig. S5. For detailed information on the TSAR program and instruction of input data preparation, please see the article (Zawadzka-Kazimierczuk A et al., J Biomol NMR 54 (2012) 81-95) or manual to the program, which can be downloaded together with the program from the website http://nmr.cent3.uw.edu.pl/software.

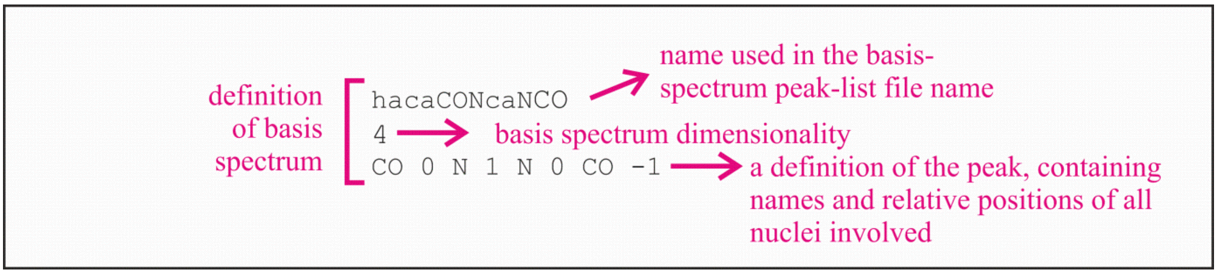

Figure S4. Explanation of the basis-experiment definition syntax for the TSAR program.

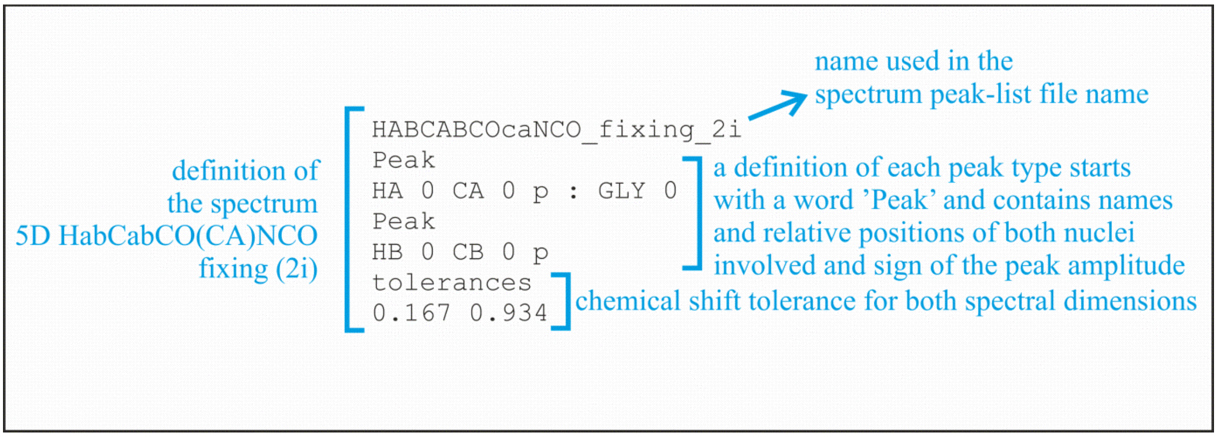

Figure S5. Explanation of other experiments definition syntax for the TSAR program.

The definition of the basis spectrum contains the peak-list name (in fact the file name should have an extension ".list", e.g. hacaCONcaNCO.list), the basis experiment dimensionality and peak definition (names and relative positions of the peak nuclei). The definition of other experiments is more complex. It contains additionally peak intensity sign and chemical shift tolerances. The sign can be positive (p), negative (n) or

unknown (u). Possible is also defining the change of the sign in the presence of glycine residue (e.g. p : GLY -1 - which means that the peak is positive unless residue i-1 is glycine). The chemical shift tolerances are typically calculated as inverse of the maximum evolution time in a given dimension, and are expressed in ppm. Below, the definitions of all the experiments with all fixing types used in this article are shown (for fixing symbols, see the article text).

basis spectrum - 4D (HACA)CON(CA)NCO:

```
hacaCONcaNCO
4
CO 0 N 1 N 0 CO -1
```

4D (HACA)CON(CA)NCO

fixing (1i):

```
hacaCONcaNCO_fixing_1i
Peak
CO -1 N 0 p : GLY -1
Peak
CO 0 N 1 n : GLY 0
tolerances
0.237 0.59
```

fixing (1ii):

```
hacaCONcaNCO_fixing_1ii
Peak
CO 0 N 1 p : GLY 0
Peak
CO 1 N 2 n : GLY 1
tolerances
0.237 0.59
```

fixing (1iii):

```
hacaCONcaNCO_fixing_1iii
Peak
N -1 CO -2 n : GLY -1
Peak
N 0 CO -1 p : GLY -1
tolerances
0.59 0.062
```

fixing (1iv):

```
hacaCONcaNCO_fixing_1iv
Peak
N 0 CO -1 n : GLY 0
Peak
N 1 CO 0 p : GLY 0
tolerances
0.59 0.062
```

5D HabCabCO(CA)NCO
fixing (2i):

```
HABCABCOcaNCO_fixing_2i
Peak
HA 0 CA 0 p : GLY 0
Peak
HB 0 CB 0 p
tolerances
0.167 0.934
```

fixing (2ii):

```
HABCABCOcaNCO_fixing_2ii
Peak
HA -1 CA -1 p : GLY -1
Peak
HB -1 CB -1 p
tolerances
0.167 0.934
```

fixing (2iii):

```
HABCABCOcaNCO_fixing_2iii
Peak
HA 0 CA 0 p : GLY 0
Peak
HB 0 CB 0 p
tolerances
0.167 0.934
```

5D HNCO(CA)NCO
fixing (3i):

```
HNCOcaNCO_fixing_3i
Peak
HN 1 N 1 p : GLY 0
tolerances
0.167 0.59
```

fixing (3ii):

```
HNCOcaNCO_fixing_3ii
Peak
HN 0 N 0 p : GLY -1
tolerances
0.167 0.59
```

fixing (3iii):

```
HNCOcaNCO_fixing_3iii
Peak
HN 1 N 1 p : GLY 0
tolerances
0.167 0.59
```

The TSAR program looks for the sequential connectivities only within a single experiment (to avoid comparison of chemical shifts originating from experiments of different resolution). Therefore, to establish the connectivities using cross-sections of a single spectrum but obtained with two different fixing types, one has to define an artificial experiment, containing the peak types of both cross-section types. For instance, on each cross-section of the 5D HNCO(CA)NCO spectrum just a single peak appears (H^N_{i+1} - N_{i+1} for (3i) and (3iii) fixing and H^N_i - N_i for (3ii) fixing), which does not allow the TSAR program to find any

sequential connectivities, even though the program has the necessary information. Thus in this case one can define a spectrum which contains both peak types. Of course there is no peak list containing both peak types. The user should create an empty peak list; the program will fill this list basing on the real peak lists. Below, the definitions of such artificial spectra for 5D HabCabCO(CA)NCO and 5D HNCO(CA)NCO experiments are shown:

```
HABCABCOcaNCO
Peak
HA -1 CA -1 u
Peak
HB -1 CB -1 u
Peak
HA 0 CA 0 u
Peak
HB 0 CB 0 u
tolerances
0.167 0.934
```

```
HNCOcaNCO
Peak
HN 0 N 0 u
Peak
HN 1 N 1 u
tolerances
0.167 0.59
```

In this article, the TSAR program was given the following input data:
data set 1 (experiments 4D (HACA)CON(CA)NCO and 5D HabCabCO(CA)NCO):
- hacaCONcaNCO (basis spectrum)
- hacaCONcaNCO_fixing_1i
- hacaCONcaNCO_fixing_1ii
- hacaCONcaNCO_fixing_1iii
- HABCABCOcaNCO_fixing_2i
- HABCABCOcaNCO_fixing_2ii
- HABCABCOcaNCO (artificial spectrum, empty peak list)

data set 2 (experiments 4D (HACA)CON(CA)NCO, 5D HabCabCO(CA)NCO and 5D HNCO(CA)NCO):
- hacaCONcaNCO (basis spectrum)
- hacaCONcaNCO_fixing_1i
- hacaCONcaNCO_fixing_1ii
- hacaCONcaNCO_fixing_1iii
- HABCABCOcaNCO_fixing_2i
- HABCABCOcaNCO_fixing_2ii
- HABCABCOcaNCO (artificial spectrum, empty peak list)
-HNCOcaNCO_fixing_3i
-HNCOcaNCO_fixing_3ii
-HNCOcaNCO_fixing(artificial spectrum, empty peak list)

The spectrum 4D (HACA)CON(CA)NCO was calculated also with fixing (1iv), but the full peak list was not prepared. This data was used just to resolve the ambiguities in case of cross-sections overlap, as shown in the article in Fig. 4.
